# Study protocol for a cluster randomised trial of sterile glove and instrument change at the time of wound closure to reduce surgical site infection in low- and middle-income countries (CHEETAH)

**DOI:** 10.1186/s13063-022-06102-5

**Published:** 2022-03-09

**Authors:** Donna Smith, Aneel Bhangu

**Affiliations:** NIHR Global Health Research Unit on Global Surgery, Institute of Translational Medicine, University of Birmingham, Heritage Building, Mindelsohn Way, Birmingham, B152TH UK

**Keywords:** Surgical site infection, Sterile gloves and instruments, Infection control, Cluster randomised trial, Study protocol, Abdominal surgery, Gastrointestinal surgery

## Abstract

**Background:**

Surgical site infection (SSI) represents a major burden for patients, doctors, and health systems around the world. The aim of this trial is to assess whether the practice of using separate sterile gloves and instruments to close wounds at the end of surgery compared to current routine hospital practice can reduce surgical site infection at 30-days post-surgery for patients undergoing clean-contaminated, contaminated, or dirty abdominal surgery.

**Methods:**

This study protocol describes a pragmatic, international, multi-centre, 2-arm, cluster randomised controlled trial, with an internal pilot. Clusters are defined as hospitals within low- and middle-income countries (LMICs) defined by the Development Assistance Committee (DAC) Official Development Assistance (ODA) list, where there are at least 4 eligible hospitals per country. Hospitals (clusters) must be in LMICs where glove and instrument change are not currently routine practice. Patients (adults and children) undergoing emergency or elective abdominal surgery for a clean-contaminated, contaminated, or dirty operation are eligible for inclusion. Before closing the abdominal wall, surgeons and the scrub nurse will change gloves and use separate, sterile instruments (intervention), versus no changing gloves or using separate, sterile instruments (standard practice, control). The primary outcome is SSI within 30 days after surgery, using the Centre for Disease Control (CDC) criteria. Secondary outcomes are SSI before point of hospital discharge, and readmission, reoperation, length of hospital stay, return to normal activities, and death up to 30-days after surgery. A 12-month internal pilot, including 12 clusters and approximately 600 participants, aims to assess adherence to allocation and follow-up of patients. The main trial is powered to detect a minimum reduction in the primary outcome from 16 to 12%. A total of 12,800 participants will be recruited from 64 clusters (hospitals) each including at least 200 participants.

**Discussion:**

Change of gloves and sterile instruments prior to fascial closure in abdominal surgery is a low-cost, simple, intraoperative intervention which involves all members of the surgical and scrub team. If effective at reducing SSI, this practice could be readily implemented across all contexts. The findings of this trial will inform future guideline updates from international healthcare organisations, including the World Health Organization.

**Trial registration:**

ClinicalTrials.gov NCT03980652. Registered on 9 July 2019

**Supplementary Information:**

The online version contains supplementary material available at 10.1186/s13063-022-06102-5.

## Administrative information

Note: the numbers in curly brackets in this protocol refer to SPIRIT checklist item numbers. The order of the items has been modified to group similar items (see http://www.equator-network.org/reporting-guidelines/spirit-2013-statement-defining-standard-protocol-items-for-clinical-trials/).

**Table:** 

Title {1}	Study protocol for a cluster randomised trial of sterile glove and instrument change at the time of wound closure to reduce surgical site infection in low- and middle-income countries (CHEETAH)
Trial registration {2a and 2b}.	ClinicalTrials.gov: NCT03980652
Protocol version {3}	Version 2.0, 9^th^ July 2019
Funding {4}	National Institute for Health Research (NIHR) Global Health Research Unit Grant (NIHR 16.136.79) and NIHR Clinical Scientist in Global Surgery personal award (Dr Aneel Bhangu; NIHR-CS-2018-18-ST2-013)
Author details {5a}	NIHR Global Health Research Unit on Global Surgery, Birmingham Clinical Trials Unit, University of Birmingham, Edgbaston, Birmingham, B15 2TH
Name and contact information for the trial sponsor {5b}	Birgit Whitman, Research Governance Team University of Birmingham Birmingham, B15 2TT researchgovernance@contacts.bham.ac.uk. The University of Birmingham Clinical Trials Unit (BCTU) is the International Coordinating Centre (ICC) for the trial. Each country will appoint a National Coordinating Investigator (NCI) and a National Coordinating Centre (the ‘Hub’) who will take responsibility for the study. The University of Birmingham is the Sponsor of the ChEETAh trial in all collaborating countries. Sponsorship will be provided by the University of Birmingham upon signing of the Clinical Trial Agreement with the Hub.
Role of sponsor {5c}	This is an investigator-initiated and investigator-led trial. The funder of the trial has no role in trial design, data collection, data analysis or data interpretation.

## Introduction

### Background and rationale {6a}

#### Background

Surgical site infection (SSI) represents a major burden for patients, doctors, and health systems around the world but is potentially preventable. SSI is the commonest postoperative complication across all income and development settings, and the commonest healthcare-associated infection in low- and middle-income countries (LMICs) [1, 2]. It has been associated with one third of postoperative deaths and accounts for 8% of all deaths caused by a nosocomial infection [3]. Rates vary significantly between different types of surgery, but it is particularly prevalent in abdominal operations with as many as one in four patients having an SSI when the operation involves the bowel [4]. SSIs cause pain and discomfort, increasing the time taken to return to work and other normal activities [5]; in resource-limited settings, this is disproportionately high since personal income is less and patients are required to pay for their own treatment [6]. The GlobalSurg-2 cohort study captured the incidence of SSI in these patients, demonstrating how it affects people around the world and how those in the poorest setting are at greatest risk [7, 8]. Whilst there are no direct data on the costs of SSI in LMICs, the burden of increased healthcare costs on patients, communities, and providers in low-income settings is likely to be substantial. Reducing SSI has an important impact on patients around the world.

#### Rationale

Improving surgical outcomes is a global health priority, highlighted by the Lancet Commission on Global Surgery [9]. Recent World Health Organization (WHO) guidelines made 29 recommendations for measures to prevent SSI, but there was little high-quality evidence to support most of these interventions [10, 11]. A Delphi consensus was undertaken by an international panel of surgeons to identify the WHO recommendations in greatest need of better supporting evidence [12]. The WHO recommendations for change of gloves and sterile instruments at the time of fascial closure were identified as the priority recommendations.

Specific evidence for SSI reduction with glove and instrument change before fascial closure is limited, including only small RCTs at high risk of bias; three studies have been published to date, all suggesting a benefit. The largest RCT (*n* = 182) included patients undergoing pancreatic surgery in a single Japanese hospital, finding a significantly lower SSI rates in the intervention group (2.2% v 12.4%, *p* = 0.002) [13]. Of the two other RCTs, the first (*n* = 92) demonstrated a reduction in the risk of SSI after vascular surgery (5.0%) versus 12.5%, *p* < 0.02) [14, 15]. These early clinical data support the need for a major, pragmatic multicentre trial in LMICs where the burden of complications is greatest.

Due to the heterogeneous evidence base, CDC, WHO, and NICE guidelines do not make recommendations on the practice of change of gloves/instruments as part of routine care. The executive summary of the 2017 WHO Guidelines on SSI reduction practice states: As there is no direct evidence of the effectiveness of sterile surgical instruments to wound closure, well-designed RCTs would be welcome. Studies should be conducted in high-, low-, and middle-income countries and include different surgical procedures using an SSI outcome defined according to CDC criteria [16].

### Objectives {7}

The aims and objectives for the pilot and the main trial are set out separately.

#### Internal pilot

The aim of the 12-month internal pilot is to assess (1) whether hospitals adhere to their allocation and (2) what proportion of patients who are eligible for ChEETAh can be followed up successfully at 30 days after their surgery.

STOP-GO criteria will be assessed at 12 months following the commencement of recruitment. A traffic light system will be used to determine feasibility of progression to the main trial:

- Green: ⩾ 80% intervention adherence in patients eligible for ChEETAh; ⩾80% of eligible patients followed up. If both criteria are met, the study will continue unchanged.
- Amber: 50–79% intervention adherence; 50–79% followed up. If at least one amber criteria is met, the study will need review to see what changes (if any) could be made for improvement.
- Red: < 50% intervention adherence; < 50% followed-up. If one or both of these criteria are met we will discuss the feasibility of the trial continuing with the Trial Steering Committee.

#### Main trial

The primary objective is to assess whether the practice of using separate, sterile gloves, and instruments to close wounds at the end of surgery can reduce surgical site infection at 30-days post-surgery for patients undergoing clean-contaminated, contaminated, or dirty abdominal surgery, compared to current routine hospital practice.

The secondary objectives are to assess the impact of changing gloves/instruments prior to wound closure compared to current routine hospital practice on secondary clinical outcomes up to 30 days post-surgery including: SSI during the in-hospital stay, re-admission, re-operation, length of hospital stay, return to normal activities, and death.

### Trial design {8}

**Fig. 1:**
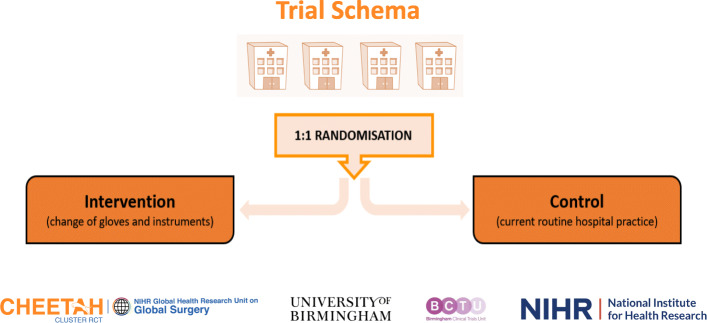
Trial schema

CHEETAH is an international, multicentre, 2-arm, cluster randomised controlled trial with an internal pilot, to evaluate the use of separate sterile gloves and instruments at the time of wound closure to reduce SSI rates in patients undergoing surgery with an abdominal incision. Clusters are defined as hospitals. Clusters will be randomly allocated (1:1) to change of gloves and instruments (intervention group) or current routine hospital practice (Fig. 1). Randomisation will be minimised by country (minimum participating of four clusters per country in order to achieve balance by treatment arm) and hospital type (referral hospital (yes or no) where a referral hospital is defined as a hospital that accepts pre-operative referrals from other surgical teams).

## Methods: participants, interventions, and outcomes

### Study setting {9}

This protocol has been reported in compliance with the Standard Protocol Items: Recommendations for Interventional Trials (SPIRIT) guideline [17].

Eligible hospitals (clusters) are any LMIC hospital performing abdominal surgery where glove and instrument change is not currently routine practice. LMICs are defined by the Development Assistance Committee (DAC) Official Development Assistance (ODA) list where there are at least 4 eligible hospitals per country. Participating sites are planned for inclusion from seven LMICs in total including (alphabetically) Benin, Ghana, India, Mexico, Nigeria, Rwanda, and South Africa.

### Eligibility criteria {10}

In clean (non-contaminated) surgery, the infection rate is low and benefits from research into interventions targeting SSI rates in those patients are limited. CHEETAH will therefore focus on clean-contaminated, contaminated, and dirty surgery.

Patients (adults and children) undergoing emergency or elective abdominal surgery for any indication (including trauma), with an intra-operative finding of clean-contaminated, contaminated, or dirty operation with at least one abdominal incision of 5 cm or greater are eligible for inclusion. Patients undergoing caesarean section will be excluded, as in many sites in LMICs, there is a risk they dominate recruitment and prevent a representative case-mix for other types of abdominal surgery. CHEETAH has no defined age limits, but each participating country will define age criteria based on country-specific regulatory approval processes.

Each participating hospital will develop a local pathway to recruit eligible patients. Potentially eligible patients can be identified by any member of the surgical team (research nurse, clinical officer, surgeon in training, operating surgeon), either before, during, or after surgery but before discharge.

### Who will take informed consent? {26a}

Individual patient-level consent for exposure to the intervention or routine hospital practice is not possible since the randomisation is at the hospital level. Hospitals randomised to the intervention are likely to change practice for all patients once randomised.

All patients undergoing abdominal surgery and who satisfy the eligibility criteria will be followed up at 30-days post-surgery. Consent for data collection in the context of the CHEETAH trial protocol will be for data collection only and will be sought at the 30-days post-surgery follow-up time point. Other data collected is collected as part of normal practice.

For patients returning as part of routine care at 30-days, written consent will be sought for the collection of the 30-day follow-up data. A patient information sheet (PIS) (see Appendix 1) will be provided to those patients providing written informed consent (see Appendix 2), following which patients are asked to give informed consent to participate in the trial. The PIS has been translated into appropriate languages as advised by local research ethics committees.

Those patients that do not return to hospital as part of standard care, will be contacted by telephone, and will be asked to provide explicit verbal consent during the call for the collection of trial-related data at 30 days, and this will be clearly documented on the CHEETAH 30-day follow-up case report form (CRF) (see Appendix 3_CHEEATH Adult CRF 30 day Follow-up_verbal consent). The consent process will be agreed by each Country Lead investigator according to relevant national guidelines and in line with country-specific regulatory board requirements.

### Additional consent provisions for collection and use of participant data and biological specimens {26b}

This trial does not involve collecting biological specimens for storage.

### Interventions

#### Explanation for the choice of comparators {6b}

The comparator is current routine hospital practice (no change of gloves or use of separate, sterile instruments *before* closing the abdominal wall).

#### Intervention description {11a}

The intervention used within CHEETAH is change of gloves and use of separate, sterile instruments before closing the abdominal wall.

In sites randomised to the intervention group, since the final level of contamination may not be known until during the operation, all cases need to be prepared for glove and instrument change, if required. Clean instruments should be set to one side if using instruments from the main tray (e.g. needle holder, forceps, suture scissor tied in a swab). If using instruments from a new pack, these should be available in the operating theatre but unopened. When to change gloves and use clean instruments: change of gloves and instruments should be undertaken for all clean-contaminated, contaminated, and dirty surgeries. After completion of the abdominal component of the operation and at the time of the count of surgical swabs and instruments (preparing for the ‘sign out’ component of the WHO Surgical Safety Checklist [18]), the surgical team should confirm eligibility of the participant.

If the patient is eligible, the surgical team should change gloves and use the clean instruments before handling the wound edges to facilitate closure. This includes two key components: (1) gloves: change of sterile gloves (or outer gloves if double gloved) for operating surgeon, assistant surgeon(s), and scrub staff (2); instruments: a sterile set of instruments should be used for abdominal wall closure including a needle holder, forceps, and scissors. This should be implemented in each hospital according to local practice and availability. For example, they can be separated from the main instruments at the start of the operation by the scrub nurse (e.g. wrapped in a clean swab). Alternatively, a new instrument(s) pack may need to be opened.

Through site surveys to address trial feasibility, most hospitals do not require additional instrument packs. Where required, Birmingham Clinical Trials Unit will work with Hubs to provide this, where feasible. The additional gloves required for the trial will be funded as part of the hospital’s normal glove purchasing processes (and then reimbursed via the Hub), to provide efficiencies in terms of supply and stock. The ICC/BCTU and the Hubs will request a report from individual hospitals for accountability of interventions.

#### Criteria for discontinuing or modifying allocated interventions {11b}

On occasion, changing gloves or instruments will occur (e.g. surgeon preference), and this will be recorded in the intraoperative CRF (see Appendix 4_Adult CRF booklet_Baseline & Intraoperative Form). Practice will be monitored throughout the trial to ensure adherence to the hospital’s allocation.

#### Strategies to improve adherence to interventions {11c}

Staff at participating hospitals (Hub or Spoke) will undergo a standardised site set-up training package. This will include (1) Online Good Clinical Practice, (2) online training modules (set-up, delivery, intervention, follow-up), (3) site initiation visit: from either BCTU or local Hub, and (4) a short ChEETAh ‘training phase’ to allow centres to adjust to change clinical practice for the delivery of the interventions. Intervention adherence will be monitored locally and centrally using the in-theatre CHEETAH Register, with the use of sterile gloves and instruments before fascial closure recorded for every case in a participating centre (see Appendix 5).

#### Relevant concomitant care permitted or prohibited during the trial {11d}

All other aspects of the operation, apart from those allocated as part of the trial intervention, will be determined by the attending surgeon and anaesthetist. Participating hospitals will implement the World Health Organization Surgical Safety Checklist [18] for all participants to standardise perioperative care prior to site opening [18]. The individual components will not be enforced.

Within this pragmatic trial, other SSI reduction measures may be used at the surgeon’s discretion (skin preparation solution, wound edge protector, triclosan sutures, wound washout, negative pressure wound therapy).

#### Provisions for post-trial care {30}

The University of Birmingham has in place Clinical Trials indemnity coverage for this trial which provides cover to the University for harm which comes about through the University’s, or its staff’s, negligence in relation to the design or management of the trial. The risk of the trial is no greater than the risk of the standard clinical care. Responsibility for the participants at sites remains with the organisation responsible for the clinical site, and it is therefore indemnified through their normal arrangements.

#### Outcomes {12}

The primary outcome is SSI up to 30-days after surgery (with the day of surgery as day 0) using the US Center for Disease Control (CDC) definition of deep incisional or superficial incisional SSI as follows:

1. The infection must occur within 30-days of the index operation;
2. The infection must involve the skin, subcutaneous, muscular or fascial layers of the incision;
3. The patient must have at least one of the following: purulent drainage from the wound; organisms detected by wound swab; diagnosis clinically or at imaging; wound opened spontaneously or by a clinician;
4. The patient has at least one of the following: pain, tenderness, localised swelling, redness, heat at the wound site, systemic fever (> 38 °C).

The secondary outcomes are:

1. SSI at discharge from hospital, based on the CDC definition;
2. Unexpected re-admission into hospital for a wound-related problem within 30-days post-surgery;
3. Unexpected re-operation for a wound-related problem within 30 days post-surgery;
4. Length of hospital stay (index hospital admission);
5. Return to normal activities within 30 days of surgery;
6. Death within 30 days of surgery

#### Participant timeline {13}

Prior to registration, final eligibility for entry into the ChEETAh trial must be confirmed. This can only be performed after the operation when the *actual* length of incision and *actual* contamination level at the time of surgery is known.

**Fig. 2:**
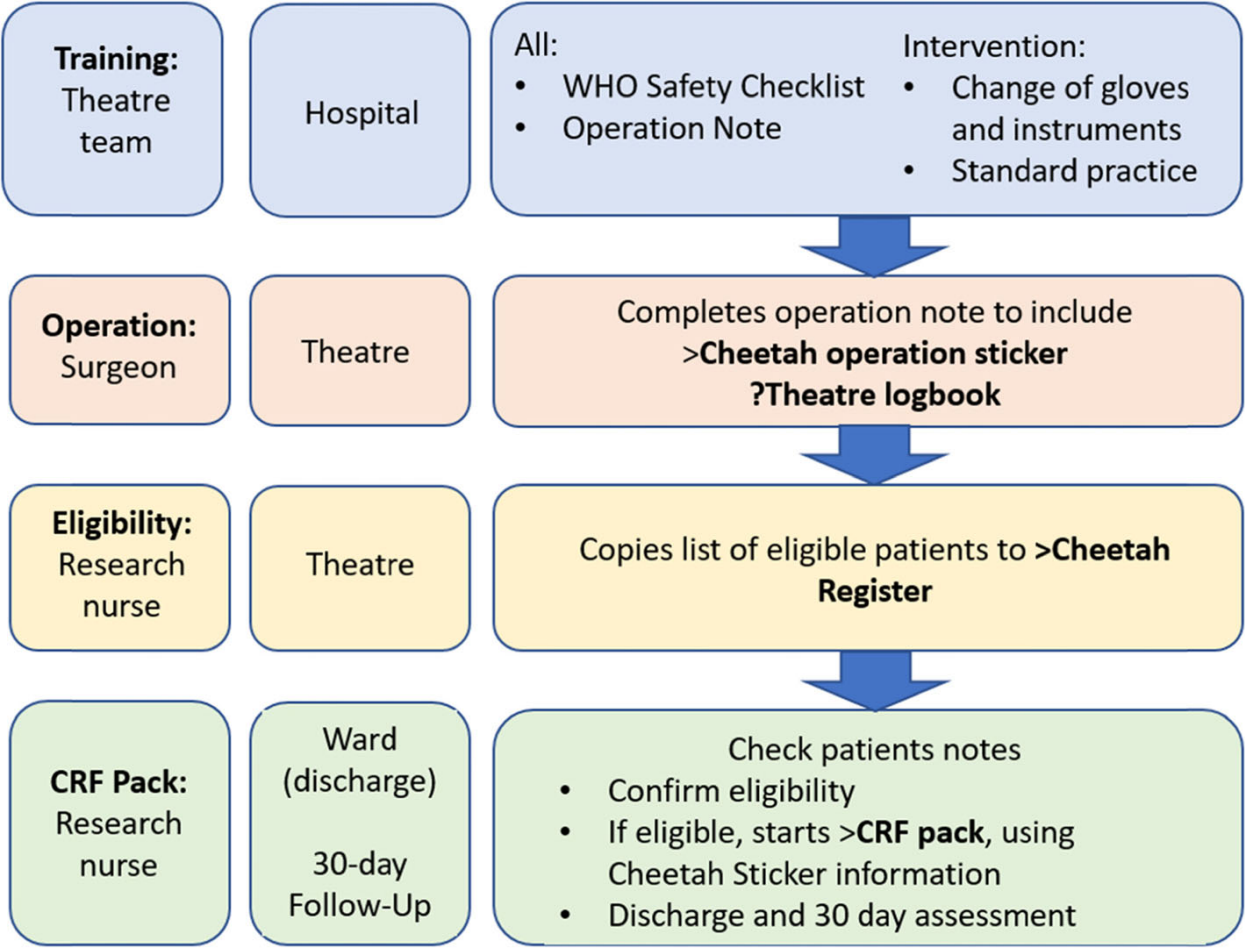
CHEETAH patient inclusion pathway

Once eligibility has been confirmed, patients will be registered by a member of the hospital-based research team. The next sheet of ChEETAh trial number stickers located in the ChEETAh Trial Investigator Site File (ISF) should be selected, along with a ChEETAh CRF booklet. The CRF booklet will capture baseline details, operative details, and SSI information at discharge (Fig. 2).

The ChEETAh trial number stickers should be affixed to the first page of the CRF booklet and all subsequent CRF pages in the spaces provided, i.e. in the box with the text ‘Please affix sticker here’. This unique trial number will be used in all correspondence between the Hub, spoke, and BCTU and will help identify patients registered in the ChEETAh trial.

The patients trial number should be recorded on the ChEETAh Trial Register and the patient should be registered on the trial database by logging on to the ChEETAh database (https://bctu-redcap.bham.ac.uk/).

Patients will be reviewed, at the time of discharge from hospital, and at post-operative day 30. If a patient achieves the primary outcome before 30 days, they should still be assessed at 30 days to record secondary outcome measures.

Once registered into the ChEETAh trial, the patient will be contacted on or after, but as close as possible to postoperative day 30 by a member of the research team (from the Hub or Spoke) to complete the 30-day follow-up assessment.

Follow-up will be completed in-person for those returning to hospital as part of standard, routine care; all other patients will be followed up by telephone 30-days post-surgery (Table 1).

**Table 1:** Participant timeline and schedule of assessments

Process	Trial document	Intraoperative	Trial entry	Discharge	30-day follow-up
Identify potential patients					
Operation	ChEETAh sticker for inclusion in operation notes)	X			
Initial eligibility check	ChEETAh register	X			
Final eligibility Check	CRF booklet ➢ Intraoperative form ➢ Patient contact form		X		
Adherence to allocated interventions	CRF booklet ➢ Intraoperative form		X		
Assessment for SSI	Follow-up form			X	X

### Sample size {14}

#### Internal pilot

Twelve centres with an average of 50 patients each during a staggered opening will result in at least 600 patients. The stop-go criteria indicates that for green light, ⩾ 80% intervention adherence in patients eligible for ChEETAh is expected (95% CI: 77% to 83%) and ⩾ 80% of eligible patients should have been followed up (95% CI: 77% to 83%). For amber light, 50–79% intervention adherence in patients eligible for ChEETAh is expected and 50–79% of eligible patients should have been followed up. For red light, < 50% intervention adherence in patients eligible for ChEETAh is expected (95% CI: 46% to 54%) and < 50% of eligible patients should have been followed up (95% CI: 46% to 54%).

#### Main trial

The sample size control group rate of 16% is based on GlobalSurg 2 feasibility data [19]. To detect an absolute difference of 4% (relative reduction of 25%) to 12%, with 90% power and using a 5% two-sided significance level, it requires 1580 participants in each group. To allow for clustering of centres the sample size was adjusted by inflating the estimate by the design effect given by 1 + (*n*–1)*ρ*, where *n* is the average cluster size and *ρ* is the estimated intra-class correlation coefficient. The intra-class correlation coefficient calculated from GlobalSurg 2 feasibility data was 0.02 [20]. We expect this to be lower in ChEETAh; there are a smaller number of centres within a limited number of countries where there is willingness to participate in a clinical trial, hence less variation is likely. With an assumption of an intra-class correlation coefficient value of 0.01, power calculations indicated that we need 30 clusters per treatment group with an average of 170 participants per cluster. After allowing for 15% loss to follow-up rate for participants and 5% drop out rate for clusters, the total sample size is 6400 per group (32 clusters per group with an average of 200 participants). This sample size also allows the cluster size to vary (coefficient of variation = 0.5). The sample size was calculated using the clustersampsi Command in Stata 15 (StataCorp LLC, TX, USA).

### Recruitment {15}

Site opening and trial recruitment will be monitored during the internal pilot phase. The ability of the CHEETAH trial delivery network’s to recruit to time and target has been demonstrated in the FALCON trial: a pragmatic multi-centre factorial randomised controlled trial testing measures to reduce surgical site infection in low- and middle-income countries [21]. This study recruited 5789 patients 12-months ahead of schedule even during the SARS-CoV-2 pandemic, demonstrating the resilience and flexibility of our research network. We will maintain flexibility to increase the number of sites or countries to reach the desired sample size if unforeseen challenges to recruitment arise, so long as the country eligibility criteria (e.g. at least 4 participating sites per country) are maintained.

### Assignment of interventions: allocation

#### Sequence generation {16a}

Clusters (hospitals) will be randomly allocated (1:1) to change of gloves and instruments (intervention group) or current routine hospital practice. Randomisation will be minimised by country (minimum participation of four clusters per country in order to achieve balance by treatment arm) and hospital type (referral hospital [yes or no]—a referral hospital is defined as a hospital that accepts pre-operative referrals from other surgical teams).

#### Concealment mechanism {16b}

Randomisation will occur centrally, and participating theatres will be confirmed in each participating centre before randomisation is performed. Hospitals will be informed of their random allocation before any patients are assessed for eligibility.

#### Implementation {16c}

The allocation sequence will be generated by the CHEETAH Trial Statistician and the site informed by the Senior Trial Manager. Selection bias will be carefully monitored by the Trial Management Group (TMG). The TMG includes those individuals responsible for the day-to-day management of the trial and will include the trial chief investigator, lead methodologists, patient representatives, and ChEETAh trial management staff from the International Coordinating Centre (ICC)/Birmingham Clinical Trials Unit (BCTU). Assessment of selection bias will primarily consist of monitoring of baseline characteristics by trial arm and by country. The aim of this will be to check for any indication that patients may have been recruited selectively (i.e. unusual or unexplained imbalance in any particular subset of patients).

In addition, during on-site visits, the ICC/BCTU will monitor Hubs, and Hubs will monitor Spokes, to confirm all eligible patients have been entered into ChEETAh by reviewing the ChEETAh register against the hospital’s standard theatre logbook. The ChEETAh register will be completed by a member of the ChEETAh research team at the hospital and is a list of all patients (compiled from the standard hospital theatre logbook). We will monitor for selection biases between arms by measuring intended and actual theatre opening plans and daily registers to confirm that all eligible patients are included.

### Assignment of interventions: blinding

#### Who will be blinded {17a}

The primary outcome of SSI will be determined by a computer-based algorithm and defined according to the CDC criteria.

Outcome assessor(s) will be trained to ask a series of specific questions relating to the definition of SSI and to record the responses provided by the patient. As this is a cluster randomised trial and outcome assessors are likely to know the hospital allocation they are unlikely to be blinded. Assessors will receive formal training in the outcome of wounds, and the computer-based algorithm will determine whether the primary outcome has been met according to the CDC criteria.

The patient will however be blinded to the treatment allocation as patients will not be aware of the cluster (hospital) randomisation and are not aware of the detailed specifics around the operation, as with any operation.

#### Procedure for unblinding if needed {17b}

As the CHEETAH intervention is a one-off in-theatre change of sterile gloves and instruments with no materials left in situ nor change to post-operative care, we do not anticipate a need to emergency unblind patients. Patients can request to be unblinded upon request after completion of the trial primary analysis.

### Data collection and management

#### Plans for assessment and collection of outcomes {18a}

Patients will be reviewed, at the time of discharge from hospital, and at post-operative day 30. All patients who have been registered and operated on regardless of adherence to the randomised hospital allocation should be followed up.

The primary outcome will be captured from the time of the index surgery until postoperative day 30. Once registered into the CHEETAH trial, the patient will be contacted on or after, but as close as possible to post-operative day 30 by a member of the research team (from the Hub or Spoke) to complete the 30-day follow-up assessment. If a patient achieves the primary outcome before 30 days, they should still be assessed at 30 days to record secondary outcome measures.

Follow-up will be completed in-person for those returning to hospital as part of standard, routine care; all other patients will be followed up by telephone 30-days post-surgery.

For patients being followed-up by telephone, they will be required to answer questions from a standardised script of questions to assess for SSI (see Appendix 6). Within the GlobalSurg-2 cohort study, 43.1% (6907/16015) of patients were approached for telephone assessment, providing initial feasibility evidence for this approach [22]. Validity of telephone wound follow-up after hospital discharge has been demonstrated in several high-quality studies. In the UK, the Bluebelle trial used telephone-based SSI follow-up for primary outcome assessment with high sensitivity and specificity [23]. A study from the USA demonstrated equivalent rates of SSI between groups undergoing telephone follow-up and in-person assessment after caesarean section [24]. In LMICs, a pre- and post-implementation study of SSI reduction measures across four sub-Saharan African countries published in *Lancet Infectious Disease* successfully used telephone follow-up of the primary outcome measure (SSI according to CDC criteria) [25]. Validation studies in Tanzania and Kenya have also demonstrated accuracy of telephone surveillance for postoperative SSI [26, 27]. Over 80% of the population of LMICs have access to a mobile telephone justifying the use of an efficient, telephone-based follow-up pathway in CHEETAH [28, 29].

In CHEETAH, a member of the site research team will contact the patient and ask the questions from the follow-up form. In the case of children, a member of the site research team will contact the parent/guardian and ask the questions from the follow-up form. SSI will be determined by a computer-based algorithm and defined according to the CDC criteria. Outcome assessor(s) will be trained to ask a series of specific questions and to record the responses provided by the patient. The algorithm will determine whether the primary outcome has been met, overcoming the subjective interpretation of CDC criteria, which has potential to introduce bias.

Other questions, as indicated, will be completed from a review of the patient notes during admission, checking hospital records (electronic or paper), discharge summaries and handover lists for re-attendances or re-admissions and checking for emergency department re-attendances.

#### Plans to promote participant retention and complete follow-up {18b}

Trial retention will be actively monitored at a site level by the ITMG. Top tips for optimising telephone follow-up and retention within global randomised trials in surgery have been co-produced between trial site investigators, research coordinators, and community engagement and involvement representatives from LMICs and will be implemented to support sites with < 95% completion of follow-up for eligible patients.

#### Data management {19}

Trial data are recorded in a variety of ways, via completion of the CHEETAH Operation Sticker (Appendix 7), CHEETAH Register, on CRFs, and then entered on to a secure online REDCap server hosted by the University of Birmingham. Data are collected intra-operatively, at trial entry, at discharge from hospital and at 30-days post-surgery (see Table 1). Source data are generally kept as part of the participants` medical notes generated and maintained at site; however, in the CHEETAH trial, the completed 30-day follow-up form will be source data. Data reported within the CRF booklet will be consistent with the source data and any discrepancies will be explained. All missing and ambiguous data will be queried. Staff delegated to complete CRFs will be trained to adhere to the requirements of data capture as explained in the ChEETAh training slides. In all cases, it remains the responsibility of the site’s local principal investigator to ensure that the CRF has been completed correctly and that the data are accurate. This will be evidenced by the signature of the site’s principal investigator, or delegate(s), on the CRF.

#### Confidentiality {27}

Data will be pseudonymised on the REDCap server (https://bctu-redcap.bham.ac.uk/) with only a unique trial number used to identify each patient, before being transferred from the site to the University of Birmingham for analysis. The security of the Trial Database System is governed by the policies of the University of Birmingham. Data management and data security within BCTU will abide by the requirements of the General Data Protection Regulations (GDPR) and any subsequent amendments. The final dataset will be stored for 25 years in accordance with UK rules.

#### Plans for collection, laboratory evaluation, and storage of biological specimens for genetic or molecular analysis in this trial/future use {33}

There will be no biological specimens collected.

## Statistical methods

### Statistical methods for primary and secondary outcomes {20a}

#### Internal pilot

Percentages of subjects that adhered to allocation and subjects that were followed up will be presented alongside 95% confidence intervals. The percentages will be presented for all centres combined and for each centre separately.

#### Main trial

A separate Statistical Analysis Plan will be produced and will provide a more comprehensive description of the planned analysis. A brief outline of the analysis is given below. All analysis will be based on the intention to treat principle, i.e. participants from all hospitals will be analysed in the groups to which the hospitals were allocated. Summary statistics will be presented for all outcome measures, with the relevant adjusted effect measures, 95% confidence intervals and *p*-values from two-sided tests. The effect of intervention will be adjusted for country and hospital type (district versus referral hospital). For all binary outcomes, adjusted relative risks (with 95% confidence intervals) will be calculated using log binomial regression models. The adjusted model will include country and hospital type as fixed effects and centre as a random effect to account for the clustered nature of the sample. If the log-binomial model fails to converge, a Poisson regression model with robust standard errors will be used to estimate the same parameters. If this also fails to converge, unadjusted estimates will be produced from the log-binomial model taking account the cluster design. It will be made clear in the final report why this occurred (e.g. not possible due to low event rate/lack of model convergence). For all time to event outcomes, Cox-proportional hazards models will be used, if the assumptions of proportionally are met, and adjusted hazard ratios with 95% confidence intervals presented. A log-rank test will be used to assess statistical significance. The primary analysis for the study will occur once all participants have completed the 30-day follow-up assessment, corresponding outcome data has been entered onto the study database, and it has been validated ready for analysis. We will conduct sensitivity analysis on imbalanced factors between trial arms, which are expected in a cluster randomised design.

### Interim analyses {21b}

Interim analyses of efficacy for presentation to the independent DMC will take place during the study. The committee will meet prior to study commencement to agree the manner and timing of such analyses but this is likely to include the analysis of the primary and major secondary outcomes at annual intervals. Criteria for stopping or modifying the study based on this information will be ratified by the DMC. Details of the agreed plan will be written into the Statistical Analysis Plan. An interim, blinded publication of the pilot study will be considered by the Trial Steering Committee to report early strategies in randomisation and allocation. By presenting these data as blinded to allocation, they can remain in the main phase analysis, and if necessary, combined event rates will be presented to prevent unblinding. Secondary publications of data will be considered by the Trial Steering Committee.

### Methods for additional analyses (e.g. subgroup analyses) {20b}

Tests for statistical heterogeneity (e.g. by including the treatment group by subgroup interaction parameter in the regression model) will be performed prior to any examination of effect estimate within subgroups. The results of subgroup analyses will be treated with caution and will be used for the purposes of hypothesis generation only. The planned subgroup analyses include country and region (West Africa, Southern Africa, India and Mexico), type of hospital (e.g. elective versus emergency), contamination of wound (e.g. clean-contamination versus contaminated/dirty), type of hospital (district versus referral), and children versus adults. We will also perform a subgroup analysis to check for consistency of treatment effect for type of consent (written versus verbal).

In addition to the main primary outcome model, a further adjusted model will be fitted to control for any baseline imbalances of key variables between the groups.

### Methods in analysis to handle protocol non-adherence and any statistical methods to handle missing data {20c}

Every attempt will be made to collect full follow-up data on all study participants using an efficient and pragmatic follow-up protocol; it is thus anticipated that missing data will be small (less than 15% loss to follow-up). Participants with missing primary outcome data will not be included in the primary analysis in the first instance. This presents a risk of bias, and sensitivity analyses will be undertaken to assess the possible impact of the risk. In brief, this may include simulating missing responses using a multiple imputation approach. Full details will be included in the Statistical Analysis Plan.

### Plans to give access to the full protocol, participant level-data, and statistical code {31c}

After the study is concluded, there are plans to share the anonymized dataset (upon submission of request to the trial chief investigator, agreement of the trial management, and completion of a data transfer agreement) and statistical code will also be made publicly available.

### Oversight and monitoring

#### Composition of the coordinating centre and trial steering committee {5d}

The University of Birmingham Clinical Trials Unit (BCTU) is the International Coordinating Centre (ICC) for the trial. Each country will appoint a National Coordinating Investigator (NCI) and a National Coordinating Centre—the Hub—who will take responsibility for the study. The Hub will take responsibility for conduct and oversight of both its own hospital and its Spoke hospitals.

The University of Birmingham is the Sponsor of the ChEETAh trial in all collaborating countries.

The Trial Management Group (TMG) includes those individuals responsible for the day-to-day management of the trial, including the trial chief investigator, lead methodologists, patient representatives, and ChEETAh trial management staff from the ICC. The group will meet approximately quarterly, but this may be more frequent if deemed necessary by the members. The role of the group is to monitor all aspects of the conduct and progress of the trial, ensure that the protocol is adhered to, and take appropriate action to safeguard participants and the quality of the trial itself. Selection bias will also be carefully monitored which primarily consists of monitoring of baseline characteristics by trial arm and by country.

The remit of the Trial Steering Committee (TSC) is to provide overall supervision of the trial and ensure that it is being conducted in accordance with the principles of Good Clinical Practice and other relevant regulations. The TSC will operate in accordance with a trial-specific TSC Charter. The specific tasks of the TSC are as follows: (i) review and approval of the trial protocol, amendments, and publications; (ii) review trial progress and advise on issues raised by other oversight committees; and (iii) review recommendations from the DMC and help with decision-making. The TSC will meet once a year (either face-to-face or via teleconferencing) or more often if required.

#### Composition of the data monitoring committee, its role and reporting structure {21a}

Data analyses will be supplied in strict confidence to an independent data monitoring committee (DMC), which will be asked to give advice on whether the accumulated data from the trial, together with the results from other relevant research, justifies the continuing recruitment of further participants. The DMC will operate in accordance with a trial specific charter based upon the template created by the Damocles Group [30]. The DMC will meet at least annually unless there is a specific reason to amend the schedule. The DMC is scheduled to meet prior to the trial commencing and at 1 year after the trial opens to recruitment and then annually thereafter until the trial closes to recruitment. Additional meetings may be called if recruitment is much faster than anticipated and the DMC may, at their discretion, request to meet more frequently or continue to meet following completion of recruitment. An emergency meeting may also be convened if required. The DMC will advise the chair of the TSC if, in their view, any of the randomised comparisons in the trial have provided proof beyond reasonable doubt that for all, or for some types of patient one particular intervention is definitely indicated or definitely contra-indicated in terms of a net difference of a major outcome. Appropriate criteria of proof beyond reasonable doubt cannot be specified precisely, but a difference of at least *p* < 0.001 (similar to a Haybittle-Peto2 stopping boundary) in an interim analysis of a major outcome may be required to justify halting, or modifying, the trial prematurely. If this criterion were to be adopted, it would have the practical advantage that the exact number of interim analyses would be of little importance, so no fixed schedule is proposed. Given the proposed use of the Haybittle-Peto boundary no adjustment for multiple testing (to control the overall type I error rate) is proposed, i.e. the threshold for statistical significance at final analysis will still be *p* = 0.05.

#### Adverse event reporting and harms {22}

As the interventions being tested in this trial are used throughout the world there are no adverse events which would be anticipated as a unique consequence of participation in the trial. No expedited reporting of adverse events is proposed. We are anticipating that there will be deaths in this trial. However, most of these deaths will be a consequence of the condition the patients presented with which leads to the need for surgery. It is possible that there may be a difference in the rate of death between the two arms of the trial if SSIs are reduced in one arm. However, this will not be detected by expedited reporting because (i) the proportion of deaths due to the trial intervention will be small compared to the background risk of death and differences will be difficult, if not impossible to detect by reporting of individual deaths, and (ii) this is a cluster RCT so adjustment for the clustering will be required to explore whether crude differences in death rates are due to the intervention. Death will be collected for all participants in the trial and this outcome will be monitored by the independent data monitoring committee.

The sponsor of the trial is responsible for notifying the regulatory bodies in writing of any serious breach of the conditions and principles of Good Clinical Practice (GCP) in connection with that of the trial or the protocol relating to that trial, within 7 days of becoming aware of that breach.

#### Frequency and plans for auditing trial conduct {23}

On-site monitoring is carried out as required following trial specific risk assessment and as documented in the monitoring plan. The monitoring of spoke hospitals will be conducted by the Hub; the Hub will be monitored by BCTU. Any monitoring activities will be reported to the central trials team at BCTU and any issues noted will be followed up to resolution. Additional on-site monitoring visits may be triggered for example by poor CRF return, poor data quality, excessive number of participant withdrawals, or deviations.

ChEETAh trial staff from BCTU will be in regular contact with the Hub research team to check on progress and address any queries that they may have. Hub trial staff will check CRFs from the Spoke hospitals for compliance with the protocol, data consistency, missing data, and timing. Hubs will send Spoke hospitals data queries for missing data or clarification of inconsistencies or discrepancies. BCTU will centrally monitor data received from the Hubs. More detailed monitoring processes will be detailed in the monitoring plan.

Local principal investigators will permit trial-related monitoring, regulatory inspections, audits, and ethical review at their site, providing direct access to source data/documents. The investigator will comply with these visits and any required follow-up. If there are any externally conducted inspections, Hubs are requested to notify BCTU in advance of any relevant inspections of the Hub site.

#### Plans for communicating important protocol amendments to relevant parties (e.g. trial participants, ethical committees) {25}

The TSC will be responsible for approving and signing off the trial protocol and any protocol amendments. Before any participants are enrolled into the trial, the PI at each site is required to obtain local approvals. Spoke sites will not be permitted to enrol participants until written confirmation of approval is received by the Hub. It is the responsibility of the PI to ensure that all subsequent amendments gain the necessary local approval. This does not affect the individual clinicians’ responsibility to take immediate action if thought necessary to protect the health and interest of individual participants. The Trial Management Group and national Hub and Spoke network leads are responsible for communicating any amendments to trial site investigators.

#### Dissemination plans {31a}

A meeting will be held after the end of the trial to allow discussion of the main results among the collaborators prior to publication. The results of the trial will be submitted for publication in a peer reviewed journal. The success of CHEETAH depends on the collaboration of a large number of clinicians across several countries. For this reason, all publications arising from this work will be attributed to the ‘Global Surgery CHEETAH Collaborative Group’, with the writing committee and order approved by the NIHR Unit of Global Surgery Executive Committee. The writing committee will include members of the International Trial Management Group (ITMG), Hub Leads and include principal investigators, and investigators who have consented or completed follow-up for a minimum of 10 patients. Any secondary publications and presentations prepared by investigators and their team members must be reviewed and approved by the Trial Management Group. Manuscripts must be submitted to the TMG in a timely fashion and in advance of being submitted for publication, to allow time for review and resolution of any outstanding issues. Authors must acknowledge that the trial was performed with the support of the University of Birmingham and funding from the NIHR. Intellectual property rights will be addressed in the clinical study site agreement between the sponsor and the National Coordinating Centre. Individuals may make a formal request for the full dataset along with a proposed statistical analysis plan. Such requests will be considered by the TMG. Although individual countries will be allowed to publish their efficacy results, the publication of efficacy results from the pooled analysis will take precedence over efficacy result publications of individual countries, unless the TMG decides otherwise.

## Discussion

CHEETAH represents one of the largest, international, cluster randomised trial in the surgical setting. The range of hospitals participating is varied and truly representative of the global network. The findings of this trial will provide evidence to influence international clinical guidelines such as the WHO recommendations on preoperative measures for SSI prevention and finally determine if the practice of change of gloves and instruments at the time of abdominal wound closure, which incur considerable costs in LMICs, reduce SSI, and will provide evidence that will impact on surgical practice everywhere. CHEETAH aims to produce high quality, generalisable data that will impact on surgical practice around the world. SSIs cause pain and discomfort, increasing the time taken to return to work and other normal activities [5]; this has an important impact on patients around the world. In resource-limited settings, this is disproportionately high since personal income is less and patients are required to pay for their own treatments and dressings [6].

A key design consideration for this trial is minimizing and monitoring for selection bias. Selection bias risks are being introduced in two ways. First is if centres change the operating theatres that they choose to collect data from after randomisation (e.g. choose not to collect data from an emergency theatre if randomised to the control arm). Secondly is if centres do not collect data on all consecutive patients undergoing surgery in pre-specified operating theatres within the hospital (cluster). We have carefully pre-specified a monitoring plan and several quality controls to mitigate against these risks. First, all centres must pre-specify the operating theatres which they will collect data from before randomisation on the Baseline Hospital Characteristics CRF (see Appendix 8). We will report this as a protocol deviation to the TMG and TSC and report this transparently in the final trial report. Secondly, we will issue a ChEETAh trial register to every participating theatre in every hospital (cluster). Every operated case in that theatre will be recorded in the theatre logbook. Eligibility is then assessed by the trained operating team, and details of all abdominal cases are included in the ChEETAh trial register, including the use (or not) of the trial intervention of change of gloves and sterile instruments for fascial closure will all be documented. This ChEETAh register can then be checked regularly against the existing in-theatre paper or electronic logbooks to monitor for selection bias. Again, these data will be regularly reviewed by the TMG and TSC.

Cluster randomised trials are common in global health but rare for evaluation of intraoperative interventions [31]. One prominent example of an in-theatre intervention applied at a cluster level was the World Health Organization Surgical Safety Checklist [18]. In this before-and-after quality improvement study, a system-level approach was taken to implementation at a hospital level, with a focus on training, local advocacy, and context adaptation to maximise adherence. Similarly, in ChEETAh, a cluster randomised design was thought to be most appropriate due to the whole-theatre team approach to implementation of change of sterile gloves and instruments at the time of fascia. During the ChEETAh design phase, the international collaboration group felt that individual randomisation would be too challenging for local teams, with high risk of contamination across groups. There is also a baseline level of sterile glove and instrument change expected in control centres (i.e. where this practice is not routine, but is indicated by the clinical situation such as gross contamination of the gloves), which would have led to difficulties in monitoring intervention adherence.

The intervention tested within ChEETAh was prioritised as part of a global consensus process [12]. Change of sterile gloves and instruments at the time of fascial closure was found not to be routine practice in the collaborating centres, and have high acceptability, with community equipoise. If a beneficial effect of change of gloves and instruments is detected in ChEETAh, the costs incurred for health systems would be low, and the intervention is likely to be cost-effective. A paired health-economic analysis of the ChEETAh trial has been preplanned and informed from data both collected within ChEETAh and from our collaborating countries in previous SSI trials [21]. Implementation has already begun as part of trial training, and this will support uptake into local policy, guidelines and practice upon reporting of the trial.

Outcome assessment is also a challenge in a trial of this magnitude. As all consecutive eligible patients enter the trial, in-person follow-up could overwhelm high-volume centres. In addition, there remain concerns around the world about risk of nosocomial SARS-CoV-2 infection in the outpatient setting. Therefore, we have opted for remote, telephone-based follow-up as the primary method of outcome assessment in ChEETAh, with a telephone interview schedule based on the US Centre for Disease Control criteria. This has precedent in the Pragmatic multi-centre factorial randomised controlled trial testing measures to reduce surgical site infection in low- and middle-income countries (FALCON) trial where 38.1% were followed-up by telephone [21].

## Trial status

This manuscript is based on CHEETAH protocol Version 2.0__09 July 2019. The first cluster opened to CHEETAH on 22 June 2020, first patient registered 24 June 2020. Estimated completion of recruitment is Q1 2022. At the time of submission, both patient recruitment and follow-up are ongoing.

## Supplementary Information

**Additional file 1: Appendix 1.** CHEETAH adult patient information sheet

**Additional file 2: Appendix 2.** ChEETAh adult consent

**Additional file 3: Appendix 3.** CHEETAH 30 day follow-up form

**Additional file 4: Appendix 4.** ChEETAh CRF booklet

**Additional file 5: Appendix 5.** ChEETAh patient register

**Additional file 6: Appendix 6.** ChEETAh telephone script

**Additional file 7: Appendix 7.** ChEETAh operation sticker

**Additional file 8: Appendix 8.** ChEETAh baseline hospital characteristics

## Abbreviations

BCTU: Birmingham Clinical Trials Unit
CDC: Centre for Disease Control
CRF: Case report form
DAC: Development Assistance Committee
DMC: Data monitoring committee
GDPR: General Data Protection Regulations
GCP: Good Clinical Practice
ICC: International Coordinating Centre
ISF: Investigator Site File
LMIC: Low- and middle-income country
NCI: National Coordinating Investigator
NIHR: National Institute for Health Research
ODA: Official Development Assistance
SSI: Surgical site infection
TMG: Trial Management Group
TSC: Trial Steering Group
WHO: World Health Organization
