# Correction: Study protocol for a cluster randomised trial of sterile glove and instrument change at the time of wound closure to reduce surgical site infection in low- and middle-income countries (CHEETAH)

**DOI:** 10.1186/s13063-022-06175-2

**Published:** 2022-04-15

**Authors:** NIHR Global Health Research Unit on Global Surgery, Adesoji O Ademuyiwa, Adewale O. Adisa, Aneel Bhangu, Peter Brocklehurst, Sohini Chakrabortee, Dhruva Ghosh, James Glasbey, Parvez D Haque, Pollyanna Hardy, Ewen Harrison, JC Allen Ingabire, Lawani Ismail, Bryar Kadir, Rachel Lillywhite, Laura Magill, Antonio Ramos de la Medina, Rachel Moore, Mark Monahan, Dion Morton, Dmitri Nepogodiev, Faustin Ntirenganya, Omar Omar, Thomas Pinkney, Donna Smith, Stephen Tabiri, Neil Winkles

**Affiliations:** Institute of Translational Medicine, University of Birmingham, Heritage Building, Mindelsohn Way, B15 2TH Birmingham, UK

**Correction to: Trials 23, 204 (2022)**

**https://doi.org/10.1186/s13063-022-06102-5**

Following the publication of the original article [1], we were notified that due to a misunderstanding during proofing, the authoring collaborator’s group was tagged as an affiliation instead of the main author.

The original article has been corrected.
