# Supplementary material for: Study protocol for a cluster randomised trial of sterile glove and instrument change at the time of wound closure to reduce surgical site infection in low- and middle-income countries (CHEETAH)
Source: Trials. 2022 Mar 9;23:204. doi: 10.1186/s13063-022-06102-5 (PMC8905008; doi:10.1186/s13063-022-06102-5)
Supplement: Supplementary file 1 — Additional file 1: Appendix 1. CHEETAH adult patient information sheet [file 13063_2022_6102_MOESM1_ESM.pdf]

CHEETAH Adult patient information sheet

Version 2.0, 30th July 2019

Background

Up to half of patients have a wound infection following surgery. Wound infections happen when germs grow in the wound causing it to become red, hot, and painful. Some wounds leak smelly pus when they are infected. This does not make you unclean, but it means you may need treatment with tablets (antibiotics). If the tablets cannot make the infection better fast enough, some people may need to have another operation. These mean more time in hospital, more expense for patients and hospitals, and longer times to get back to work or school.

Doctors do several things to reduce the chances of wound infection. One of the things they can do is to change their gloves and the surgical instruments used during the operation, *before* closing the tummy.

CHEETAH Study

The purpose of our study is to test whether using separate sterile gloves and instruments to close the wound at the end of the operation compared to not changing gloves and instruments during the operation can reduce the chances of wound infection. This study only looks at surgical wound(s) on the tummy area of the body and not anywhere else. To allow a fair comparison in the study of the different techniques, your hospital will be 'randomly' allocated which method will be used for your operation. You may not be made aware of which method was used for your operation.

Standard practice is for theatre staff not to change their gloves or instruments during the operation, this is known to be very safe. What we are trying to find out is, if gloves and instruments are changed during the operation, before closing the tummy area does this reduce the chances of patients getting a wound infection.

What this study entails

This study will include only very minimal changes to your treatment. The decision about which method is to be used during your operation will be decided by a computer, so that there is a fair spread of people and hospitals across the different groups.

Time commitment

The time commitment for you is very low. Whilst in hospital we will have collected some routinely held data from your hospital notes about your operation and your wound following the surgery, and at your 30-day follow up visit we will ask you some extra questions, although these will only take a few minutes.

Collecting study results

To collect the results for the study, our team will ask you some questions at your 30-day visit about your recent operation and your wound and some questions about how you have been since you were discharged from hospital.

Information to be collected

Only simple information about you, your operation, and how it affects you will be collected. This will include your name, but you will only ever be viewed by your patient number. We will keep this information separate from your address.

Confidentiality

Information about you will be kept confidential. Information collected about you that is needed for the research will be sent to the University of Birmingham (England) which is coordinating this study. This information will be stored for 25 years after the end of the trial but will then be destroyed. We will keep the data as safely as possible and with as few details as possible.

Who is organising and funding the research?

The CHEETAH trial is being coordinated by University of Birmingham (England), which is also the sponsor for the trial. CHEETAH is funded by the National Institute for Health Research (NIHR), which is based in England.

What if there is a problem?

Taking part in the study would not affect your legal rights. If you are harmed by taking part in this research project, University of Birmingham will be liable if harm was due to the research and not the underlying clinical care. If the harm is due to someone's negligence, then you may have grounds for a legal action but you may have to pay for this. Whether or not you take part in the study, if you wish to complain, or have any concerns about any aspect of the way you have been approached or treated during the course of this study, you should ask to speak to the researchers involved in the study who will do their best to answer your questions (contact details are at the bottom of this form).

Consent

It is up to you to decide to join the study. If you agree to take part, you will be agreeing to the collection and transfer of information about your operation and your wound following the operation. We will ask you to sign (or fingerprint) a consent form. You are free to leave the study at any time, without giving a reason. This would not affect the care you receive.

Right to withdraw

You can withdraw your consent for the data collected at the 30-day follow-up appointment to be used in the trial, at any time, up until the time when we analyse the data at the end of the study. The estimated date of analysis is June 2021.

Local PI name *Local PI address*
Hub name *Hub address* *Hub telephone*
