# Supplementary material for: Study protocol for a cluster randomised trial of sterile glove and instrument change at the time of wound closure to reduce surgical site infection in low- and middle-income countries (CHEETAH)
Source: Trials. 2022 Mar 9;23:204. doi: 10.1186/s13063-022-06102-5 (PMC8905008; doi:10.1186/s13063-022-06102-5)
Supplement: Supplementary file 2 — Additional file 2: Appendix 2. ChEETAh adult consent [file 13063_2022_6102_MOESM2_ESM.pdf]

CHEETAH Consent Form (Adult)

Version 1.0, 17th July 2019

This is a consent form for the collection and transfer of data within the CHEETAH trial.

	CHEETAH Trial Number	Please affix sticker here							
	Centre Name								
	Patient Date of Birth	d	d	m	m	y	y	y	y

#	**Statement (please read)**	**Patient** *(initial or thumb print each box)*
1	I have read/ had read to me the Patient Information Sheet for the above study (version ____) and have had the opportunity to consider the information and ask questions.	
2	I understand that my participation in this study is voluntary and that I may withdraw at any time, without giving a reason. This will not affect my medical care, or legal rights.	
3	I understand that a copy of the consent form and information about me will be supplied in confidence to study coordinators and that this information will be transferred to University of Birmingham (UK), for use in the CHEETAH trial.	
4	I understand that my medical notes may be looked at by the research team, the Sponsor or by regulatory bodies examining research practice. I give permission for these individuals to have access to my records.	
5	I understand that my consent for CHEETAH is consent to the collection and transfer of data relating to my operation and wound.	
6	I understand that 30 days after my operation, the study researchers will ask me questions about my operation and wound.	
7	I understand I am free to withdraw my consent for the data collected at the 30-day visit to be used in the trial, at any time, up until the time when the data is analysed at the end of the study.	
8	I agree to take part in the above study and for this to be recorded in my personal health record.	
9	I also agree to information about me related to the study being stored on a password protected computer system.	

**Name of patient**								**Name of doctor (or research staff) taking consent**							
**Signature (or thumb print) of patient**								**Signature of doctor (or research staff)**							
**Date form signed (or thumb printed) by patient:**								**Date form signed by doctor (or research staff):**							
d	d	m	m	y	y	y	y	d	d	m	m	y	y	y	y
