# Supplementary material for: Study protocol for a cluster randomised trial of sterile glove and instrument change at the time of wound closure to reduce surgical site infection in low- and middle-income countries (CHEETAH)
Source: Trials. 2022 Mar 9;23:204. doi: 10.1186/s13063-022-06102-5 (PMC8905008; doi:10.1186/s13063-022-06102-5)
Supplement: Supplementary file 3 — Additional file 3: Appendix 3. CHEETAH 30 day follow-up form [file 13063_2022_6102_MOESM3_ESM.pdf]

CHEETAH CLUSTER RCT | NIHR Global Health Research Unit on Global Surgery

BCTU Birmingham Clinical Trials Unit

CHEETAH 30 DAY FOLLOW-UP FORM

	CHEETAH Trial Number	Please affix sticker here
	Centre name	____________________
	Date of Birth (month/year)	m m y y y y

Before asking patients any trial-related questions at this 30-day follow-up, patients must have provided explicit verbal informed consent. By completing and signing this form you are confirming the patient provided verbal consent (during the 30-day follow-up contact) for the collection of their data.

Follow-up details

Date of follow-up	d d m m y y y y

Patient status

Has patient died?	☐ Yes	☐ No
If patient died, date of death	d d m m y y y y	

If patient is still in hospital at this 30-day time-point, please complete the 30-day Follow-up Form in hospital, by speaking directly with the patient

Consent

Has patient provided verbal consent (at the time of the 30-day follow-up contact) for the collection and transfer of the 30-day follow-up data? *(if no/declined please complete the `form completed by` section at the bottom of this form)*	☐ Yes	☐ No/declined
**If yes, date patient provided verbal consent**	d d m m y y y y	

Follow-Up Questions

Since discharge from hospital following surgery, has the patient returned to normal activities for example; school, work or family duties?	☐ Yes	☐ No

From the day of surgery up until 30-days post-operatively: have there been any of the following at the abdominal wound (skin, subcutaneous, muscle and fascia layers):
NB: If the patient has died, were any of the following present at the abdominal wound prior to death?

Pain or tenderness at the wound?	☐ Yes	☐ No
Localised swelling around the wound?	☐ Yes	☐ No
Redness of the wound?	☐ Yes	☐ No
Heat at the wound site?	☐ Yes	☐ No
Pus draining from the wound?	☐ Yes	☐ No
Fever?	☐ Yes	☐ No
Has the patient been re-admitted to hospital?	☐ Yes	☐ No

Has the patient been re-operated on?	☐ Yes	☐ No
If yes, was the re-operation for SSI?	☐ Yes	☐ No
**The remainder of the information on this form should be checked from hospital records:**		
**Up until 30-days post-operatively:**		
Are abdominal wound swab results available?	☐ Yes	☐ No
If yes, were any pathological organism(s) identified from a specimen from the superficial incision or subcutaneous tissue?	☐ Yes	☐ No
Was abdominal wound opening present (spontaneously opened or by clinician)?	☐ Yes	☐ No
Was SSI diagnosed by clinician or on imaging?	☐ Yes	☐ No
How has follow-up been performed? *(tick all that apply)*	☐ Phone call<br>☐ In-person, community<br>☐ In-person, hospital<br>☐ Clinical notes	

**Form completed by**									
Print full name									
Signature	Date form completed	d	d	m	m	y	y	y	y
