# Supplementary material for: Study protocol for a cluster randomised trial of sterile glove and instrument change at the time of wound closure to reduce surgical site infection in low- and middle-income countries (CHEETAH)
Source: Trials. 2022 Mar 9;23:204. doi: 10.1186/s13063-022-06102-5 (PMC8905008; doi:10.1186/s13063-022-06102-5)
Supplement: Supplementary file 4 — Additional file 4: Appendix 4. ChEETAh CRF booklet [file 13063_2022_6102_MOESM4_ESM.pdf]

CHEETAH CLUSTER RCT | NIHR Global Health Research Unit on Global Surgery

BCTU Birmingham Clinical Trials Unit

CHEETAH

CASE REPORT FORMS

(30-day verbal consent)

RCT-2

ClustEr randomised Trial of sterile glove And instrument change at closure to reduce surgical site infection

ChEETAh Trial Number	Please affix sticker here							
Centre name								
Date of Birth	d	d	m	m	y	y	y	y

Is the patient participating in the FALCON Trial? Yes ☐ No ☐

If yes please provide the FALCON trial number ☐☐☐☐☐☐

Or not known ☐

CHEETAH CLUSTER RCT | NIHR Global Health Research Unit on Global Surgery

BCTU Birmingham Clinical Trials Unit

CHEETAH PATIENT CONTACT FORM

	CHEETAH Trial Number	Please affix sticker here
	Centre name	
**Patient name**		
First name(s)		
Last name(s)		
**Contact numbers**		
Landline phone number		
Mobile phone number(1)		
Mobile phone number(2)		
**Other contact details (1)**		
Contact name		
Relationship to patient		
Phone number		
**Other contact details (2)**		
Contact name		
Relationship to patient		
Phone number		

**Form completed by**											
Print full name											
Signature		Date form completed	d	d	m	m	y	y	y	y	

CHEETAH CLUSTER RCT | NIHR Global Health Research Unit on Global Surgery

BCTU Birmingham Clinical Trials Unit

CHEETAH BASELINE AND INTRAOPERATIVE FORM

	CHEETAH Trial Number	Please affix sticker here
	Centre name	______________________
	Date of Birth (month/year)	m m y y y y

BASELINE		
Patient Age	☐☐ years	
Gender	☐ Female	☐ Male
Does the patient have known diabetes?	☐ Yes	☐ No
Does the patient have known HIV status?	☐ Known negative<br>☐ Status not known	☐ Known positive
What is the patient`s smoking status?	☐ Ex-smoker (stopped more than 6 weeks ago)<br>☐ Current smoker or stopped less than 6 weeks ago	☐ Never smoked
**Operation details**		
Date of operation	d d m m y y y y	
Timing of surgery	☐ Elective	☐ Emergency (unplanned)
Indication for surgery	☐ Malignant disease<br>☐ Trauma	☐ Benign disease
WHO Surgical safety checklist used?	☐ Yes	☐ No
American Society of Anesthesiologists grade *(definitions overleaf)*	☐ Grade I<br>☐ Grade III<br>☐ Grade V	☐ Grade II<br>☐ Grade IV
Was intraoperative pulse oximetry used?	☐ Yes	☐ No
Up to 60 minutes prior to incision, were prophylactic antibiotics given?	☐ Yes	☐ No
Hair removal at site of wound?	☐ In theatre – electric<br>☐ In theatre – razor/blade<br>☐ Before theatre arrival	☐ Not applicable (no hair at site of wound)<br>☐ Not done
What was the operative approach?	☐ Open – midline<br>☐ Laparoscopic	☐ Open – non-midline<br>☐ Laparoscopic converted to open
Largest abdominal incision greater than or equal to 5cm?	☐ Yes	☐ No
**Actual** intra-operative contamination *(definitions overleaf)*	☐ Clean<br>☐ Contaminated	☐ Clean-contaminated<br>☐ Dirty

Main abdominal operation performed	please name: ______________________________ ______________________________
**CHEETAH INTERVENTION**	
Did all surgeon(s) and scrub nurse(s) involved in wound closure, change gloves ***BEFORE*** closing the abdominal wall?	☐ No<br>☐ Yes
Did all surgeon(s) and scrub nurse(s) involved in wound closure, use separate, sterile instruments ***BEFORE*** closing the abdominal wall?	☐ No<br>☐ Yes

**Form completed by**										
Print full name										
Signature		Date form completed	d	d	m	m	y	y	y	y

CHEETAH CLUSTER RCT | NIHR Global Health Research Unit on Global Surgery | BCTU Birmingham Clinical Trials Unit

CHEETAH INTRAOPERATIVE FORM DEFINITIONS

American Society of Anesthesiologists physical status classification system (modified for CHEETAH)	
**Grade**	**Definition**
**I**	A normal healthy patient
**II**	A patient with mild systemic disease
**III**	A patient with severe systemic disease
**IV**	A patient with severe systemic disease that is a constant threat to life
**V**	A moribund patient who is not expected to survive without the operation

Contamination of surgery anticipated at time of registration

	**Do not include** ⇩	**Include** ⇩	**Include** ⇩	**Include** ⇩
	**Clean**	**Clean-contaminated**	**Contaminated**	**Dirty**
**Definition**	GI/ GU tracts not entered	GI/GU tracts entered	Minor spillage of contents of GI/GU tracts	Gross spillage of contents of GI/GU tracts, or established peritonitis
**Typical urgency of surgery**	Elective	Elective/Emergency	Elective/Emergency	Emergency
**Example procedures**<br>***Please note, list is NOT exhaustive***	• Hernia repair (no bowel resection anticipated)<br>• Adhesiolysis	• Appendicectomy (non-inflamed appendix anticipated)<br>• Cholecystectomy (no bile spillage anticipated)<br>• Bowel resection (no gross spillage of faeces anticipated)	• Appendicectomy (inflamed, non-perforated appendix anticipated)<br>• Laparotomy for infarcted or necrotic bowel (no perforation anticipated)	• Laparotomy for perforated duodenal ulcer<br>• Hartmann's procedure for perforated sigmoid colon<br>• Old traumatic wounds with devitalized tissue

The above list is not exhaustive and is intended to provide a small number of examples only

If the predicted contamination changes, for example during surgery the wound is defined as a `clean wound`, any collected data will not be used for analysis and can be destroyed appropriately

CHEETAH CLUSTER RCT | NIHR Global Health Research Unit on Global Surgery | BCTU Birmingham Clinical Trials Unit

CHEETAH FOLLOW-UP FORM AT DISCHARGE

To be completed by doctor or research nurse at the time of patient discharge from hospital.
For questions with tick boxes, please tick one box per question.

	CHEETAH Trial Number	Please affix sticker here
	Centre name	______________________
	Date of Birth (month/year)	m m y y y y

**Follow-up details**		
Has patient died prior to hospital discharge?	☐ Yes	☐ No
If patient died, date of death	d d m m y y y y	
If patient did not die, date of discharge from hospital	d d m m y y y y	
**From the day of surgery up until discharge (or death):**		
Did patient have purulent drainage from the abdominal wound?	☐ Yes	☐ No
Are abdominal wound swab results available?	☐ Yes	☐ No
If yes, were any pathological organism(s) identified from a specimen from the superficial incision or subcutaneous tissue?	☐ Yes	☐ No
Was abdominal wound opening present (spontaneously opened or by clinician)?	☐ Yes	☐ No
Was SSI diagnosed by clinician or on imaging?	☐ Yes	☐ No
Did patient have systemic fever (greater than 38 degrees Celsius)?	☐ Yes	☐ No
**From the day of surgery up until discharge have there been any of the following at the abdominal wound (skin, subcutaneous, muscle and fascia layers)? NB: If the patient died, were any of the following present at the abdominal wound prior to death?**		
Was there pain or tenderness at the wound?	☐ Yes	☐ No
Was there localised swelling around the wound?	☐ Yes	☐ No
Was there redness of the wound?	☐ Yes	☐ No
Was there heat at the site of the wound?	☐ Yes	☐ No

**Form completed by**			
Print full name			
Signature		Date form completed	d d m m y y y y

CHEETAH CLUSTER RCT | NIHR Global Health Research Unit on Global Surgery | BCTU Birmingham Clinical Trials Unit

CHEETAH 30 DAY FOLLOW-UP FORM

	CHEETAH Trial Number	Please affix sticker here
	Centre name	______
	Date of Birth (month/year)	m m y y y y

Before asking patients any trial-related questions at this 30-day follow-up, patients must have provided explicit verbal informed consent. By completing and signing this form you are confirming the patient provided verbal consent (during the 30-day follow-up contact) for the collection of their data.

**Follow-up details**	
Date of follow-up	d d m m y y y y
**Patient status**	
Has patient died?	☐ Yes ☐ No
If patient died, date of death	d d m m y y y y

If patient is still in hospital at this 30-day time-point, please complete the 30-day Follow-up Form in hospital, by speaking directly with the patient

**Consent**		
Has patient provided verbal consent (at the time of the 30-day follow-up contact) for the collection and transfer of the 30-day follow-up data? *(if no/declined please complete the `form completed by` section at the bottom of this form)*	☐ Yes	☐ No/declined
**If yes, date patient provided verbal consent**	d d m m y y y y	

**Follow-Up Questions**		
Since discharge from hospital following surgery, has the patient returned to normal activities for example; school, work or family duties?	☐ Yes	☐ No

From the day of surgery up until 30-days post-operatively: have there been any of the following at the abdominal wound (skin, subcutaneous, muscle and fascia layers):
NB: If the patient has died, were any of the following present at the abdominal wound prior to death?

Pain or tenderness at the wound?	☐ Yes	☐ No
Localised swelling around the wound?	☐ Yes	☐ No
Redness of the wound?	☐ Yes	☐ No
Heat at the wound site?	☐ Yes	☐ No
Pus draining from the wound?	☐ Yes	☐ No
Fever?	☐ Yes	☐ No
Has the patient been re-admitted to hospital?	☐ Yes	☐ No

Has the patient been re-operated on?	☐ Yes	☐ No
If yes, was the re-operation for SSI?	☐ Yes	☐ No
**The remainder of the information on this form should be checked from hospital records:**		
**Up until 30-days post-operatively:**		
Are abdominal wound swab results available?	☐ Yes	☐ No
If yes, were any pathological organism(s) identified from a specimen from the superficial incision or subcutaneous tissue?	☐ Yes	☐ No
Was abdominal wound opening present (spontaneously opened or by clinician)?	☐ Yes	☐ No
Was SSI diagnosed by clinician or on imaging?	☐ Yes	☐ No
How has follow-up been performed? *(tick all that apply)*	☐ Phone call<br>☐ In-person, community<br>☐ In-person, hospital<br>☐ Clinical notes	

**Form completed by**									
Print full name									
Signature	Date form completed	d	d	m	m	y	y	y	y
