# Supplementary material for: Study protocol for a cluster randomised trial of sterile glove and instrument change at the time of wound closure to reduce surgical site infection in low- and middle-income countries (CHEETAH)
Source: Trials. 2022 Mar 9;23:204. doi: 10.1186/s13063-022-06102-5 (PMC8905008; doi:10.1186/s13063-022-06102-5)
Supplement: Supplementary file 5 — Additional file 5: Appendix 5. ChEETAh patient register [file 13063_2022_6102_MOESM5_ESM.pdf]

CHEETAH Register

Patient name	Date and time of operation<br><br>*dd/mm/yyyy hh:mm*	Date of birth	Sex	Name of abdominal operation	Post-operative ward	At least one abdominal incision greater than or equal to 5cms?	Actual intraoperative contamination: clean-contaminated, contaminated, or dirty?	Age appropriate (according to local ethics requirements)?	Eligible to participate in the CHEETAH trial?	If eligible, affix CHEETAH Trial Number
Abcde Fghijk	*04/09/2019 14:30*	*30/04/1988*	*Female*	*Appendicectomy*	*Ward 522*	☑ Yes<br>☐ No	☑ Yes<br>☐ No	☑ Yes<br>☐ No	☑ Yes<br>☐ No	CHEETAH CLUSTER RCT NIHR Global Health Research Unit on Global Surgery<br>ChEETAh Trial Number<br>999999
						☐ Yes<br>☐ No	☐ Yes<br>☐ No	☐ Yes<br>☐ No	☐ Yes<br>☐ No	*Affix Trial Number if eligible*
						☐ Yes<br>☐ No	☐ Yes<br>☐ No	☐ Yes<br>☐ No	☐ Yes<br>☐ No	*Affix Trial Number if eligible*
						☐ Yes<br>☐ No	☐ Yes<br>☐ No	☐ Yes<br>☐ No	☐ Yes<br>☐ No	*Affix Trial Number if eligible*
						☐ Yes<br>☐ No	☐ Yes<br>☐ No	☐ Yes<br>☐ No	☐ Yes<br>☐ No	*Affix Trial Number if eligible*
						☐ Yes<br>☐ No	☐ Yes<br>☐ No	☐ Yes<br>☐ No	☐ Yes<br>☐ No	*Affix Trial Number if eligible*
