# Supplementary material for: Study protocol for a cluster randomised trial of sterile glove and instrument change at the time of wound closure to reduce surgical site infection in low- and middle-income countries (CHEETAH)
Source: Trials. 2022 Mar 9;23:204. doi: 10.1186/s13063-022-06102-5 (PMC8905008; doi:10.1186/s13063-022-06102-5)
Supplement: Supplementary file 6 — Additional file 6: Appendix 6. ChEETAh telephone script [file 13063_2022_6102_MOESM6_ESM.pdf]

CHEETAH CLUSTER RCT | NIHR Global Health Research Unit on Global Surgery

BCTU Birmingham Clinical Trials Unit

CHEETAH 30 DAY FOLLOW UP: TELEPHONE SCRIPT

Purpose of the telephone script

To provide a standardised tool for the collection of the 30-day follow-up data via telephone contact with the patient and to minimise any subjective interpretation of the assessment of Surgical Site Infection (SSI)

This script must be used in conjunction with the CHEETAH 30-day Follow-up Form

General guidance on the telephone script and completion of the 30-day Follow-up Form

- Prior to contacting the patient ensure you have completed the tasks listed below in the section marked: `Before contacting the patient`
- Pay close attention to the text in the section marked: `Consent`
- Telephone the patient and follow the script provided
- Text in **bold black** text (in the script) is text directly for the patient, i.e should be spoken to the patient
- Text in *black italics* (in the script) are <u>instructions</u> for the outcome assessor performing the 30-day Follow-up contact
- Text in *red italics* (in the script) can be adapted locally

Before contacting the patient:

Affix the CHEETAH trial number sticker, to the CHEETAH 30-day Follow-Up Form, in the box as indicated and enter the Centre Name and patient's date of birth.

Enter the date of follow-up in the space provided and indicate how the follow-up has been performed i.e (phone call, in-person [hospital], in-person [community], clinical notes) (ticking all that apply)

Next confirm the status of the patient i.e answer the question: `has the patient died` tick one box (Yes or No).

If the patient has died, enter the date of death in the space provided. Please complete the follow-up questions (other than the question related to return to normal activities), from the hospital notes, to confirm if any of the symptoms were present at the abdominal wound prior to death.

Consent

Before asking patients any trial-related questions at this 30-day follow-up contact, patients must have provided explicit verbal informed consent. Document the consent on the 30-day Follow-up Form.

<u>Note:</u> By completing and signing the 30-day Follow-up Form you are confirming the patient provided verbal consent during the 30-day telephone call for the collection of their data.

TELEPHONE SCRIPT

Telephone the patient *and follow the script below:*

**`Hello, my name is** *<name of person making the call>*, **Please may I speak to** *<insert* patient's *name>*`. *Follow either Scenario 1 or Scenario 2 whichever applies.*

Scenario 1: If the patient *is unavailable or unable to take the call:*

**`When would it be a good time to call back and speak to** *<insert* patient's *name>***directly?** `

Await response

`Thank you very much, I will call back at that time`.

Scenario 2: If you are speaking directly to the patient*:*

`I am contacting you from *<insert name of hospital/institution>* **regarding your recent operation. I would like to ask you a few questions about your operation and your wound and some questions about how you have been since you were discharged from hospital. It will take about 5 minutes.**

Before we continue, the information we collect from you today is being used as part of an international research study called CHEETAH we are running here at *<insert name of hospital/institution>* **and in lots of other countries throughout the world. The study is being run from the University of Birmingham in the UK, specifically to look at how we can reduce the chances of patients getting a wound infection following surgery. Any of the information you provide today will be kept confidential and will be stored securely and only the people directly involved in the study will have access to this data.`**

`You can withdraw consent for the data collected during this call, at any time, up until the results are analysed`

`If you agree to continue with the call, you will be agreeing to the collection and transfer of information to the University of Birmingham about your operation and your wound following the operation`.

`Are you happy to provide your verbal consent to continue and answer a few questions?`

>>>If the patient *declines to continue, thank them for their time and politely end the call*
>>>If the patient *provides verbal consent to continue this MUST be documented.*

(Document the patient *`s response to the question on the CHEETAH 30 DAY FOLLOW UP Form)*

Follow-Up Questions

Ask the patient *each of the questions as detailed below. Tick one box per question according to the answers provided by the* patient*, and insert the* patient *`s response on the 30-Day Follow up Form in the Case Report Form booklet.*

Start with the question:

`Since discharge from hospital following surgery, have you returned to normal activities for example; school, work or family duties?`

`Yes`
`or No`
(record the response on the 30-day Follow-up Form)

`For the following questions could you please answer either yes or no. From the day of surgery up until today, have you had any of the following symptoms`:

`Pain or tenderness at the wound?` *(record the response on the 30-day Follow-up Form)*

`any localised swelling around the wound?` *(record the response on the 30-day Follow-up Form)*

`Any redness of the wound?` *(record the response on the 30-day Follow-up Form)*

`Has the wound felt hot to touch?` *(record the response on the 30-day Follow-up Form)*

**\`Has there been any pus or fluid draining from the wound?\`** *(record the response on the 30-day Follow-up Form)*<br><br>**\`Have you had a temperature or fever?\`** *(record the response on the 30-day Follow-up Form)*<br><br>**\`Have you been re-admitted to hospital?\`** *(record the response on the 30-day Follow-up Form)*<br><br>**\`Have you had another operation? \`** *(record the response on the 30-day Follow-up Form) If the patient replies <u>yes</u> to this question ask the patient* **Was the re-operation for a wound infection? \`***(record the response on the 30-day Follow-up Form)*<br><br>**\`That is the end of the questions I had for you today. Thank you so much for taking the time to speak with me today, we very much appreciate it. Thank you and goodbye\`**
At the end of the call, the remaining questions on the 30-day Follow-up Form should be completed from the patient\`s hospital records. Record which method was used i.e (responses directly from the patient or via hospital records) and record this on the 30-day Follow-Up Form in the space provided.
**This is the end of the 30-day Follow-Up assessment**
