# Supplementary material for: Study protocol for a cluster randomised trial of sterile glove and instrument change at the time of wound closure to reduce surgical site infection in low- and middle-income countries (CHEETAH)
Source: Trials. 2022 Mar 9;23:204. doi: 10.1186/s13063-022-06102-5 (PMC8905008; doi:10.1186/s13063-022-06102-5)
Supplement: Supplementary file 7 — Additional file 7: Appendix 7. ChEETAh operation sticker [file 13063_2022_6102_MOESM7_ESM.pdf]

CHEETAH CLUSTER RCT | NIHR Global Health Research Unit on Global Surgery

# CHEETAH OPERATION STICKER

**This patient may be eligible to participate in the ChEETAh trial.**

**Please complete the following information IN THEATRE AND affix this sticker to the operation/hospital notes:**

| | | |
|---|---|---|
| WHO surgical safety checklist used? | ☐ Yes | ☐ No |
| Was intraoperative pulse oximetry used? | ☐ Yes | ☐ No |
| Pre-incision prophylactic antibiotics given (within 60 minutes of incision)? | ☐ Yes, antibiotic ______________ | ☐ No |
| Hair removal at site of wound? | ☐ In theatre - electric<br>☐ In theatre - razor/blade<br>☐ Before theatre arrival | ☐ Not applicable (no hair at site of wound)<br>☐ Not done |
| Largest abdominal incision ≥ 5cms? | ☐ Yes | ☐ No |
| Actual intra-operative contamination | ☐ Clean<br>☐ Contaminated | ☐ Clean - contaminated<br>☐ Dirty |
| Did all surgeon(s) and scrub nurse(s) involved in wound closure, change gloves **BEFORE** closing the abdominal wall? | ☐ Yes | ☐ No |
| Did all surgeon(s) and scrub nurse(s) involved in wound closure, use separate, sterile instruments **BEFORE** closing the abdominal wall? | ☐ Yes | ☐ No |


CHEETAH CLUSTER RCT | NIHR Global Health Research Unit on Global Surgery

CHEETAH OPERATION STICKER

This patient may be eligible to participate in the ChEETAh trial.

Please complete the following information IN THEATRE AND affix this sticker to the operation/hospital notes:

| | | |
|---|---|---|
| WHO surgical safety checklist used? | ☐ Yes | ☐ No |
| Was intraoperative pulse oximetry used? | ☐ Yes | ☐ No |
| Pre-incision prophylactic antibiotics given (within 60 minutes of incision)? | ☐ Yes, antibiotic ______________ | ☐ No |
| Hair removal at site of wound? | ☐ In theatre - electric<br>☐ In theatre - razor/blade<br>☐ Before theatre arrival | ☐ Not applicable (no hair at site of wound)<br>☐ Not done |
| Largest abdominal incision ≥ 5cms? | ☐ Yes | ☐ No |
| Actual intra-operative contamination | ☐ Clean<br>☐ Contaminated | ☐ Clean - contaminated<br>☐ Dirty |
| Did all surgeon(s) and scrub nurse(s) involved in wound closure, change gloves **BEFORE** closing the abdominal wall? | ☐ Yes | ☐ No |
| Did all surgeon(s) and scrub nurse(s) involved in wound closure, use separate, sterile instruments **BEFORE** closing the abdominal wall? | ☐ Yes | ☐ No |



CHEETAH CLUSTER RCT | NIHR Global Health Research Unit on Global Surgery

CHEETAH OPERATION STICKER

This patient may be eligible to participate in the ChEETAh trial.

Please complete the following information IN THEATRE AND affix this sticker to the operation/hospital notes:

| | | |
|---|---|---|
| WHO surgical safety checklist used? | ☐ Yes | ☐ No |
| Was intraoperative pulse oximetry used? | ☐ Yes | ☐ No |
| Pre-incision prophylactic antibiotics given (within 60 minutes of incision)? | ☐ Yes, antibiotic ______________ | ☐ No |
| Hair removal at site of wound? | ☐ In theatre - electric<br>☐ In theatre - razor/blade<br>☐ Before theatre arrival | ☐ Not applicable (no hair at site of wound)<br>☐ Not done |
| Largest abdominal incision ≥ 5cms? | ☐ Yes | ☐ No |
| Actual intra-operative contamination | ☐ Clean<br>☐ Contaminated | ☐ Clean - contaminated<br>☐ Dirty |
| Did all surgeon(s) and scrub nurse(s) involved in wound closure, change gloves **BEFORE** closing the abdominal wall? | ☐ Yes | ☐ No |
| Did all surgeon(s) and scrub nurse(s) involved in wound closure, use separate, sterile instruments **BEFORE** closing the abdominal wall? | ☐ Yes | ☐ No |



CHEETAH CLUSTER RCT | NIHR Global Health Research Unit on Global Surgery

CHEETAH OPERATION STICKER

This patient may be eligible to participate in the ChEETAh trial.

Please complete the following information IN THEATRE AND affix this sticker to the operation/hospital notes:

| | | |
|---|---|---|
| WHO surgical safety checklist used? | ☐ Yes | ☐ No |
| Was intraoperative pulse oximetry used? | ☐ Yes | ☐ No |
| Pre-incision prophylactic antibiotics given (within 60 minutes of incision)? | ☐ Yes, antibiotic ______________ | ☐ No |
| Hair removal at site of wound? | ☐ In theatre - electric<br>☐ In theatre - razor/blade<br>☐ Before theatre arrival | ☐ Not applicable (no hair at site of wound)<br>☐ Not done |
| Largest abdominal incision ≥ 5cms? | ☐ Yes | ☐ No |
| Actual intra-operative contamination | ☐ Clean<br>☐ Contaminated | ☐ Clean - contaminated<br>☐ Dirty |
| Did all surgeon(s) and scrub nurse(s) involved in wound closure, change gloves **BEFORE** closing the abdominal wall? | ☐ Yes | ☐ No |
| Did all surgeon(s) and scrub nurse(s) involved in wound closure, use separate, sterile instruments **BEFORE** closing the abdominal wall? | ☐ Yes | ☐ No |
