# Supplementary material for: Study protocol for a cluster randomised trial of sterile glove and instrument change at the time of wound closure to reduce surgical site infection in low- and middle-income countries (CHEETAH)
Source: Trials. 2022 Mar 9;23:204. doi: 10.1186/s13063-022-06102-5 (PMC8905008; doi:10.1186/s13063-022-06102-5)
Supplement: Supplementary file 8 — Additional file 8: Appendix 8. ChEETAh baseline hospital characteristics [file 13063_2022_6102_MOESM8_ESM.pdf]

CHEETAH

Baseline Hospital Characteristics

CASE REPORT FORM

ClustEr randomised Trial of sterile glove And instrument change at closure to reduce surgical site infection

Hospital name	
Country	

This form should be completed during the site set-up phase to ensure the baseline hospital characteristics are an accurate reflection of characteristics before a site is randomised.

CHEETAH CLUSTER RCT | NIHR Global Health Research Unit on Global Surgery | BCTU Birmingham Clinical Trials Unit

BASELINE CHARACTERISTICS	
Referral hospital? (accepts referrals from other hospitals, e.g. University Hospital)	☐ Yes<br>☐ No
How many theatres does the hospital have?	Total number ☐☐☐
Which patients are you likely to include in the CHEETAH trial?<br>(*tick all that apply*)	☐ Upper GI/Hepatobiliary<br>☐ Colorectal surgery<br>☐ Gynaecology<br>☐ Urology<br>☐ Other *(please list):* ____________<br>☐ Emergency<br>☐ Elective
Which theatres will you include in the CHEETAH trial?	☐ All theatres in the hospital<br>☐ Only selected theatres (please list): ____________
Approximately how many abdominal operations are performed in these CHEETAH theatres each week?	☐☐☐ Per week
**Practice**	
Agreed minimum age of eligible CHEETAH participants in your hospital	☐☐ years
For all abdominal operations, is it routine practice for all surgeon(s) and scrub nurse(s) involved in wound closure, to ***change gloves BEFORE*** closing the abdominal wall?	☐ Yes<br>☐ No
For all abdominal operations, is it routine practice for all surgeon(s) and scrub nurse(s) involved in wound closure, to ***use separate, sterile instruments BEFORE*** closing the abdominal wall?	☐ Yes<br>☐ No
Is it routine practice to prescribe prophylactic antibiotics prior to predicted contaminated abdominal surgery?	☐ Yes<br>☐ No
Is it routine practice to use alcoholic chlorhexidine skin prep prior to midline laparotomy?	☐ Yes<br>☐ No
Are you likely to enter the same participants at your hospital into both CHEETAH and the FALCON trial?	☐ Yes<br>☐ No

**Form completed by**										
Print full name										
Signature		Date form completed	d	d	m	m	y	y	y	y
